# Sex and population differences in the cardiometabolic continuum: a machine learning study using the UK Biobank and ELSA-Brasil cohorts

**DOI:** 10.1186/s12889-024-19395-9

**Published:** 2024-08-06

**Authors:** Daniela Polessa Paula, Marina Camacho, Odaleia Barbosa, Larissa Marques, Rosane Harter Griep, Maria Jesus Mendes da Fonseca, Sandhi Barreto, Karim Lekadir

**Affiliations:** 1 National School of Statistical Sciences, Brazilian Institute of Geography and Statistics, Rio de Janeiro, Brazil; 2 Institute of Mathematics and Statistics, University of the Rio de Janeiro State, Rio de Janeiro, Brazil; 3 Departament de Matemàtiques i Informàtica, Universitat de Barcelona, Barcelona, Spain; 4 Institute of Nutrition, University of the Rio de Janeiro State, Rio de Janeiro, Brazil; 5 Coordination of Information and Communication (CINCO - PEIC), Oswaldo Cruz Foundation, Rio de Janeiro, Rio de Janeiro, Brazil; 6 Postgraduate Program in Public Health, School of Medicine & Clinical Hospital, Federal University of Minas Gerais, Belo Horizonte, Brazil; 7 National School of Public Health, Oswaldo Cruz Foundation, Rio de Janeiro, Brazil; 8 Health and Environmental Education Laboratory, Oswaldo Cruz Institute (IOC), Rio de Janeiro, RJ Brazil

**Keywords:** Cardiometabolic continuum, Cardiometabolic trajectories, Machine learning, SHAP, UK Biobank, ELSA-Brasil

## Abstract

**Background:**

The temporal relationships across cardiometabolic diseases (CMDs) were recently conceptualized as the cardiometabolic continuum (CMC), sequence of cardiovascular events that stem from gene-environmental interactions, unhealthy lifestyle influences, and metabolic diseases such as diabetes, and hypertension. While the physiological pathways linking metabolic and cardiovascular diseases have been investigated, the study of the sex and population differences in the CMC have still not been described.

**Methods:**

We present a machine learning approach to model the CMC and investigate sex and population differences in two distinct cohorts: the UK Biobank (17,700 participants) and the Brazilian Longitudinal Study of Adult Health (ELSA-Brasil) (7162 participants). We consider the following CMDs: hypertension (Hyp), diabetes (DM), heart diseases (HD: angina, myocardial infarction, or heart failure), and stroke (STK). For the identification of the CMC patterns, individual trajectories with the time of disease occurrence were clustered using k-means. Based on clinical, sociodemographic, and lifestyle characteristics, we built multiclass random forest classifiers and used the SHAP methodology to evaluate feature importance.

**Results:**

Five CMC patterns were identified across both sexes and cohorts: EarlyHyp, FirstDM, FirstHD, Healthy, and LateHyp, named according to prevalence and disease occurrence time that depicted around 95%, 78%, 75%, 88% and 99% of individuals, respectively. Within the UK Biobank, more women were classified in the Healthy cluster and more men in all others. In the EarlyHyp and LateHyp clusters, isolated hypertension occurred earlier among women. Smoking habits and education had high importance and clear directionality for both sexes. For ELSA-Brasil, more men were classified in the Healthy cluster and more women in the FirstDM. The diabetes occurrence time when followed by hypertension was lower among women. Education and ethnicity had high importance and clear directionality for women, while for men these features were smoking, alcohol, and coffee consumption.

**Conclusions:**

There are clear sex differences in the CMC that varied across the UK and Brazilian cohorts. In particular, disadvantages regarding incidence and the time to onset of diseases were more pronounced in Brazil, against woman. The results show the need to strengthen public health policies to prevent and control the time course of CMD, with an emphasis on women.

**Supplementary Information:**

The online version contains supplementary material available at 10.1186/s12889-024-19395-9.

## Introduction

Cardiometabolic diseases (CMDs) are the leading causes of mortality and morbidity worldwide and present a challenge for health systems [1]. CMDs are not isolated diseases but rather a spectrum of conditions deeply inter-related, which include diabetes, stroke, and cardiovascular diseases (CVDs) [1]. The recently defined concept of the cardiometabolic continuum (CMC) highlights the importance of considering the temporal relationships between CMDs for the development of prevention strategies [1–3]. Based on the cardiovascular continuum definition, the CMC provides a broader perspective, acknowledging that the progression of CMDs can be traced along a trajectory of chained events referred to as a continuum, where each event signifies the manifestation of a new disease [1–3].

Although the pathophysiological pathways linking metabolic and cardiovascular diseases have been investigated, including their variations by sex, current studies on CMDs have not considered a multi-disease longitudinal approach. Instead, the prevailing modeling strategies for studying CMDs resolve around isolated diseases, disease counts, sectional CMDs patterns, or merely examining the order of occurrence, regardless of the disease [4–6]. In this sense, machine learning techniques represent a promising tool for discovering complex patterns of relationships not found by traditional methods. To the best of our knowledge, to date, no studies have been conducted to identify longitudinal patterns of the CMC and assess potential variations across sexes and diverse populations [4–6].

Studies have consistently revealed sex differences in CMDs and their risk factors, suggesting that men tend to experience hypertension at earlier stages and have a higher prevalence of cardiometabolic multimorbidity [7–9]. On the other hand, research focusing on individuals with diabetes show distinct risks for CVDs, with women exhibiting a higher risk of developing myocardial infarction, heart failure, and ischemic heart disease than men [8, 9]. Furthermore, the significance and relative impact of risk factors can vary between sexes. For instance, in the context of CVDs, age, hypertension, total cholesterol, and low-density lipoprotein (LDL) are prominent risk factors for men, while, diabetes, triglyceride levels, and high-density lipoprotein (HDL) cholesterol affect women more [10, 11].

Additionally, cross-country variations in CMDs and risk factors prevalence trends have been reported. In the last years, low- and middle-income countries (LMICs) experienced increasing deaths due to CVDs, a faster increase rate of diabetes, and a higher prevalence of CVDs in younger populations when compared to high-income countries (HICs) [7, 12]. Moreover, more pronounced sex disparities regarding CMDs treatment and prevention were observed in LMICs [13]. These differences, potentially enhanced by cultural, environmental and health system differences, suggest that CMC and their dissimilarities by sex require population-specific analyses, which can lead to distinct patterns between LMICs and HICs. Thus, understanding these variations with population-specific analyses, as conducted in our study, becomes crucial when implementing effective prevention strategies and public health policies tailored to each population’s needs [7, 12, 13].

It is well established that intervening at any stage within the continuum of events as envisioned in the CMC can effectively modify the progression of CMDs [1–3]. However, these interventions pose a challenge to conventional medical practices, which typically focus on treating diseases in isolation and are often not tailored to specific sexes [14]. Therefore, identifying the pattern of evolution of CMC and exploring the differences between sexes, populations, and individual risk factors may help in the development of integrated and personalized health policies for prevention and treatment, aiming to change the time course of CMDs. Thus, the objective of this study is to identify sex differences in CMC patterns by using machine learning techniques and ascertain the relative importance of individual risk factors. Furthermore, by leveraging the UK Biobank and ELSA-Brasil cohorts, we aim to understand whether sex differences are consistently shared between two distinct populations (UK and Brazil) characterized by large ethnic and socioeconomic diversity.

## Methods

### Study population

Two large cohorts were used in this study: the UK Biobank and ELSA-Brasil. The UK Biobank is a based-population prospective health study that included a first assessment conducted between 2006 and 2010 for 502,640 participants (40–69 years) and holds a linkage to routine healthcare data. More details about the study design can be seen in previous publications [15]. Participants, at least 40 years of age, with no previous history at baseline of hypertension, diabetes, cardiovascular disease (myocardial infarction, angina, and heart failure), and stroke, with at least 10 years of follow-up and two assessments were included in this study, totaling 17,700 participants.

The ELSA-Brasil is a multi-center cohort study conducted in six Brazilian capitals. The main objective is to investigate the incidence and risk factors associated with cardiovascular diseases and diabetes. The baseline was performed from 2008 to 2010 for 15,105 active or retired civil servants (35–74 years). The study design of ELSA-Brasil was reported previously [16, 17]. Participants, at least 40 years old, with no previous history at the baseline of hypertension, diabetes, cardiovascular disease (myocardial infarction, angina, or heart failure), and stroke, with at least 10 years of follow-up, and four assessments were included in this study, totaling 7162 participants. Flowcharts for both study populations are available in the Appendix (SFig 5, and SFig 6).

The UK Biobank had approval granted by the NHS National Research Ethics Service (generic ethics approval for, approval letter dated 17 June 2011, Ref 11/NW/0382). ELSA-Brasil was approved by the National Research Ethics Committee (Conep—No. 976), and the research protocol developed at all Research Centers was approved by the Research Ethics Committee of the Oswaldo Cruz Institute (CEP Fiocruz/IOC—No. 343/06). All participants gave written consent to participate.

### Variables

#### Outcome variables

Based on the CMC description, the following cardiometabolic diseases were included: hypertension, diabetes (diabetes mellitus), heart diseases (angina, myocardial infarction, or heart failure), and stroke. For each participant, the CMC trajectory was defined as the set containing the occurrence times of each one of the diseases considered, in terms of the fraction of the follow-up time. More specifically, we consider the 4-tuple (t_hypertension_, t_diabetes_, t_heart_disease_, t_stroke_), where the time of occurrence of the disease *i* assumes a value t_i_, such that 0 < t_i_ ≤1, taking value 1 when the disease does not occur during the follow-up. For example, if a participant in a 10 year follow-up period reported hypertension after 2 years, diabetes after 4 years, and reported no other disease, the cardiometabolic continuum is identified by the 4-tuple (0.2, 0.4,1.0,1.0). In this way, it is possible to maintain the temporal ordering necessary for the identification of the CMC patterns and to avoid biases due to unbalanced data.

The conditions in the UK Biobank were identified using the International Classification of Disease (ICD-10) codes from linkage to Hospital Episode Statistics (HES). Participants were considered to have a disease if they had valid International Classification of Diseases (ICD)-10-CM codes from a hospital episode occurring before the date of the assessment center. More details and ICD codes of the diseases are available in the supplementary material (Appendix S1, SChart 1). The conditions in the ELSA-Brasil, such as angina, myocardial infarction, heart failure, and stroke were identified by the participant’s self-report of a previous diagnosis by a physician. Hypertension was defined by systolic blood pressure ≥ 140 mm Hg and/or diastolic blood pressure ≥ 90 mm Hg and/or antihypertensive treatment [18, 19]. Diabetes was defined by self-report or use of medication. When not reported, it was defined by clinical information from fasting plasma glucose (≥ 126 mg/dL; 7.0 mmol/L), 2-h plasma glucose during the Oral Glucose Tolerance Test 75 g (> 200 mg/dL; 11.1 mmol/L), or glycated hemoglobin (≥ 6.5%; 48 mmol/mol) [19, 20].

#### Predictors variables

The set of covariates selected in this study was based on previously published studies on CMDs and related factors [8–12]. We considered the covariates that did not imply additional costs beyond those easily accessible by physicians during a clinical visit, including age (in years), sex (male, female), smoking status (never, past, current), and a set of anthropometric and clinical variables: body mass index (BMI) (kg/m2), waist-hip ratio, systolic blood pressure (BP) (mmHg), diastolic BP (mmHg), heart rate (BPM), cholesterol, glucose, LDL, HDL, glycated hemoglobin (HbA1c), triglycerides were similarly defined for both cohorts. Maternal education (never attended school, incomplete elementary, elementary, secondary, and undergraduate) and family history of diseases (yes, no): hypertension, diabetes, heart disease, stroke, sudden death, were defined only for the ELSA-Brasil cohort. Past smoking (most/all days, occasionally, tried/twice, never), and coffee type (decaffeinated, instant, ground, another type) were defined only for the UK Biobank cohort [21–25]. Chart 1 presents the definition of other socioeconomic, lifestyle and dietary variables [21–25]. All the covariates were assessed at baseline.

**Chart 1:** Socioeconomic and lifestyle and dietary variables according to cohort

Sociodemographic			Lifestyle and diet		
	UK Biobank	ELSA-Brasil		UK Biobank	ELSA-Brasil
**Education**	None, secondary (A/AS, O/GCSEs, CSE and NVQ/NHD/NHC), and university/ professional (university, college, and professional)	Elementary (never attended school to elementary, secondary (high school to incomplete university), university	**Alcohol**	Never, past, and current	Not consuming, moderate (< 210 and 140 g/wk for men and women, respectively), and excessive ( higher than previous limits)
**Ethnicity**	White, not white (Mixed, Asian, Black, Chinese, and other ethnic groups)	White, not white (“Pardo,” Black, Indigenous, and Asian)	**Physical activity**	Duration of moderate/vigorous activity (minutes usually spend doing moderate/vigorous activities on a typical day)	Weak, moderate, and strong (following the IPAQ’s classification)
**Marital status/ Live with others**	Live with others in the main home (yes, no)	Marital status (single, not single)	**Sleeping problem**	Never/rarely, usually, sometimes	Yes, no
**Income/ Townsendeprivation**	Townsendeprivation index	Per capita household income (dolar)	**Fruit consumption and vegetable/salad consumption**	Number of pieces/tablespoons usually per day	High (twice or more per day), daily (once a day/five to six times a week), weekly (two to four times a week), and rarely (once a week or less)
			**Coffee consumption**	Yes, no	No, yes, with caffeine, yes, decaffeinated

### Statistical analysis

All analyses were stratified by sex (men/woman) and population (UK/Brazil). The incidence and average time of occurrence of each chronic condition were also analyzed. Participants who developed a given condition were grouped by the number of diseases developed during follow-up, and differences between groups were evaluated by Pearson’s Chi-square test, analysis of variance, and the Kruskal-Wallis test.

The k-means clustering technique was used to identify the CMC patterns. Participants were clustered according to their CMC trajectories. The average silhouette method was applied to determine the number of clusters. To describe the CMC patterns represented by each cluster, the incidence and average time of occurrence of each disease were considered, as well as the order of occurrence of the diseases in the CMC trajectories classified in the cluster. Sex differences were evaluated by Pearson’s Chi-square test, analysis of variance, and the Kruskal-Wallis test. Further details can be seen in Appendix S1, SMethods 1.

The multiclass random forest classifier was implemented considering as an outcome the CMC patterns identified by the clusters. The classifier was chosen based on the best performance obtained when the data is imbalanced [19]. Due to the observed imbalance between clusters, the weighted performance measures (accuracy, precision, recall, and F1-score) were estimated by the average of stratified 10-fold cross-validation (CV), repeated 5 times. In each iteration, the algorithms were trained on nine partitions and evaluated on the remaining partition. We consider 5-fold nested cross-validation for hyperparameter tuning, most appropriate for a trustworthy evaluation of performance measures. The procedures of imputation, preprocessing (standardization of numerical variables and one hot encoder for categorical variables), and synthetic minority over-sampling technique (SMOTE), on the training partitions only, necessary to guarantee the quality and consistency of the data, and to build a reliable and robust classifier, were performed within the CV, on the test and training partitions separately, to avoid bias in performance estimation. Technical details are given in Appendix S1, SMethods 2.

To provide interpretability of the classifiers, we use the SHapley Additive exPlanations (SHAP) approach, a powerful tool for extracting the importance of variables for classification, as well as the directionality of their effect [26]. SHAP can simultaneously identify both local and global interpretability features. This means it enables to determine the global importance of features for prediction and to evaluate how this influence changes depending on the magnitude of the values (lower or higher) observed in the features (local importance) [27]. The SHAP values were calculated within the CV algorithm.

The software R 4.3.0 was used for descriptive analysis, statistical tests, and building clusters by k-means. Python 3.9 was used for building classifiers. The level of statistical significance was set at *p* < .05.

## Results

A total of 17,700 participants from the UK Biobank with a mean follow-up time of 10.9 years, (sd = 0.6) and 7162 from the ELSA-Brasil with a mean follow-up time of 12.3 years (sd = 0.7), were included in this study. The mean age for the participants was 55.1 years (sd = 7.6, range 40–71) in the UK Biobank, and 50.8 (sd = 7.4, range of 40–75) in the ELSA-Brasil. In terms of sex distribution, 9242 (52.2%) and 4292 (59.9%) were women in the UK and Brazilian cohorts, respectively. It is worth noting that two cohorts differed in their ethnic distributions, with 16,126 (91.1%) white participants in the UK Biobank against 4132 (57.2%) white participants in the ELSA-Brasil, reflecting the considerable socio-demographic heterogeneity between the two countries. In the UK Biobank, 1693 participants (9.6%) had one disease during follow-up, 349 (2.0%) had two, and 35 (0.2%) had three diseases during follow-up. As for the ELSA-Brasil participants, 2314 participants (32.3%) had one disease during follow-up, 490 (6.8%) had two, and 49 (0.7%) had three diseases. The sociodemographic characteristics of the study populations are described in Appendix S1, STable 1.

Table 1 presents the incidence and average time of disease occurrence. Among women in the ELSA-Brasil cohort, 1740 (40.5%) developed some disease during follow-up, with 35.1% developing hypertension, 8% diabetes, 4.4% heart disease, and 0.8% stroke. Among men, 1113 (38.8%) reported some disease, with 33.1% developing hypertension, 7% diabetes, 6.1% heart disease, and 1.2% stroke. Diabetes and stroke occurred faster in women than in men, and heart diseases had a higher incidence among men. Regarding the UK Biobank, a significantly higher percentage of men developed some disease during follow-up (1260, 14.9% of men versus 817, 8.8% of women). Among women, 7.4% had hypertension, 1.4% diabetes, 1.1% heart disease, and 0.2% stroke, whereas, for men, the incidence was higher for all diseases (12%, 2.2%, 3.4%, and 0.6% for hypertension, diabetes, heart disease, and stroke, respectively).

**Table 1:** Incidence and average occurrence time (in terms of follow-up fraction) of diseases, according to the sex and cohort

UK Biobank (*N* = 17,700)					
Men (*N* = 8458)			Woman (*N* = 9242)		
CMD	N, (%)	Time Mean (SD)	CMD	N, (%)	Time Mean (SD)
HYP	1018 (12)	0.43 (0.23)	HYP	683* (7.4)	0.44 (0.23)
DM	194 (2.3)	0.46 (0.23)	DM	133* (1.4)	0.43 (0.22)
HD	290 (3.4)	0.45 (0.23)	HD	106* (1.1)	0.46 (0.23)
STK	49 (0.6)	0.51 (0.21)	STK	23* (0.2)	0.47 (0.21)
**ELSA - Brasil (** ***N*** ** = 7162)**					
Men (*N* = 2870)			Woman (*N* = 4292)		
CMD	N, (%)	Time, mean (SD)	CMD	N, (%)	Time, mean (SD)
HYP	950 (33.1)	0.46 (0.27)	HYP	1510 (35.1)	0.46 (0.26)
DM	202 (7)	0.62 (0.25)	DM	343 (8)	0.55^+^ (0.27)
HD	174 (6.1)	0.51 (0.29)	HD	191* (4.4)	0.51 (0.27)
STK	36 (1.2)	0.62 (0.29)	STK	35 (0.8)	0.48^+^ (0.29)

^*^Significant incidence difference between men and women. ^+^ Significant time difference between men and women. CMD: Cardiometabolic Diseases, HYP: Hypertension, DM: Diabetes Mellitus, HD: Heart Diseases, and STK: Stroke

The incidence and mean time of occurrence according to the sex and number of conditions developed during follow-up are presented in Appendix S1, STable 2. Within the UK Biobank, the incidence of hypertension and heart disease is higher among men than women, either as standalone or with other diseases, while diabetes and stroke occur more frequently associated with other diseases among men. For the ELSA-Brasil, the isolated incidence of heart disease is higher among men, while the isolated incidence of diabetes and hypertension is higher among women. Furthermore, diabetes occurred faster among women who developed multiple diseases than among men.

**Fig. 1:**
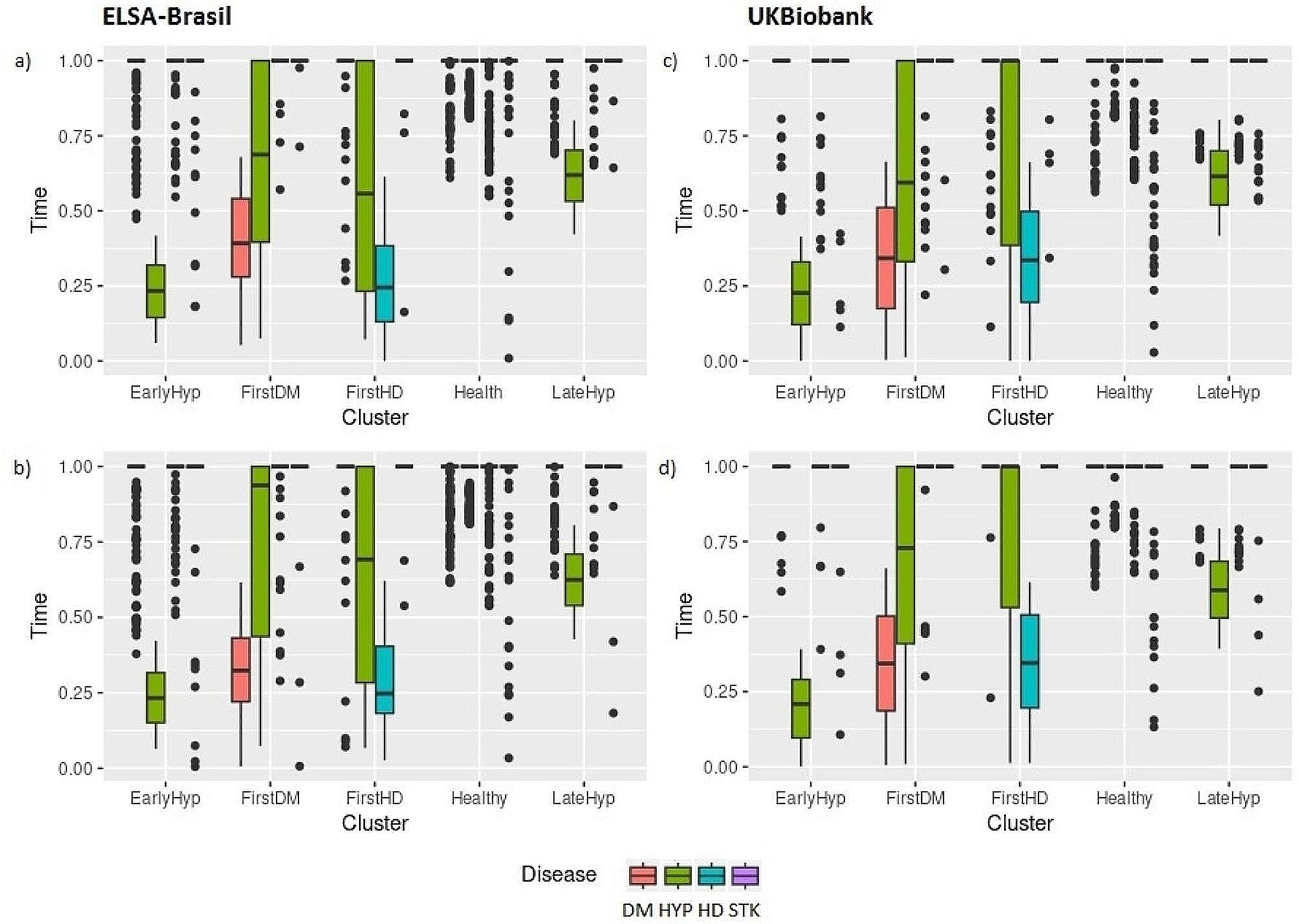
Distribution of disease occurrence times (in terms of follow-up fraction) among clusters for ELSA-Brasil (**a**) men and (**b**) women, and UKBiobank (**c**) men and (**d**) women. DM: Diabetes Mellitus, HYP: Hypertension, HD: Heart Disease, STK: Stroke

For the identification of CMC patterns, the participants were grouped into clusters according to their CMC trajectories, in terms of the 4-tuples with the occurrence fraction times. Overall, the measures for evaluating the number of clusters showed five clusters as the optimal number of partitions for both sexes, regardless of the cohort. Figure 1 presents the distributions of disease occurrence times in clusters.

Similarities can be observed among the four analysis scenarios depicted in Fig. 1, denoted by (a) male in the ELSA-Brasil cohort, (b) female in the ELSA-Brasil cohort, (c) male in the UKBiobank cohort, and (d) female in the UKBiobank cohort. Each of these scenarios has two clusters where all participants reported a fraction time for hypertension less than 1, thus all had hypertension during follow-up, with the difference that in one cluster the condition appears earlier than the other. Additionally, there is a distinct cluster where all participants reported heart disease, another where all reported diabetes, and finally, one cluster where the majority remained healthy (with only outliers representing participants who reported diseases at follow-up). The similarities found in the distributions of occurrence times, coupled with those observed in the incidences and disease trajectories reported in Appendix S1, STable 2, and Table 2, have enabled the classification of five general patterns of CMC, EarlyHyp, FirstDM, FirstHD, Healthy, and LateHyp, named according to prevalence and disease occurrence time, and encompassing the four scenarios analyzed:

i) EarlyHyp - Hypertension (Hyp) is the first disease to occur on the continuum (for at least 95% of participants in the cluster). All participants in the cluster had hypertension, which occurred up to 40% of the time at follow-up.
ii) LateHyp - Hypertension is the first disease to occur on the continuum (for at least 92% of participants in the cluster). All participants in the cluster had hypertension, which occurred in the interval from 40 to 80% of the follow-up.
iii) FirstHD - Heart Disease (HD) is the first disease to occur on the continuum for at least 75% of cluster participants in scenarios 1 (a) and b), and 90% of participants in scenarios 1 c) and d). All participants had heart disease, which occurred within 60% of the follow-up time for scenarios 1 a), (b) and d), and within 70% of the follow-up time for scenario 1 c).
iv) FirstDM - Diabetes Mellitus (DM) is the first disease to occur on the continuum for at least 78% of cluster participants in scenarios 1 a) and b), and 94% of participants in scenarios 1 (c) and d). All participants in the cluster had diabetes, which occurred from 10 to 70% of the follow-up time for scenario 1 a), up to 60% of the follow-up time for scenario 1 b), and up to 70% of the time for scenarios 1 c) and d).
v) Healthy - Most participants did not develop diseases during follow-up for at least 88% of the participants in the cluster in scenarios 1 a) and b), and 98% of the participants in scenarios 1 c) and d). A lower percentage of participants developed diseases, usually in isolation, and later than other clusters.

STable 3, in Appendix S1, shows the incidence and time of occurrence of diseases in the clusters. In the ELSA-Brasil cohort, more men (69%, *n* = 1981) were allocated to the Healthy cluster compared to women (66.1%, *n* = 2837). In the Healthy cluster, a small percentage of participants developed diseases, usually later and in isolation, and the percentage of men who developed heart disease was higher than for women (52, 2.6% versus 41, 1.4%). More women were allocated to the FirstDM cluster (167, 3.9% of women, versus 81, 2.8% of men), and the time to the occurrence of diabetes was lower (0.32 vs. 0.38). In the UK Biobank cohort, a contrasting pattern emerged, as there were more women in the Healthy cluster (8490, 91.8% of women versus 7313, 86.4% of men), and a higher representation of men in the other clusters where participants reported at least one disease. Similar to the ELSA-Brasil cohort’s Healthy cluster, in the UKBiobank, a small number of participants in this cluster had a given disease, typically occurring later and in isolation. Additionally, the percentage of those who developed diabetes, heart disease, and stroke was higher among men than among women within the Healthy cluster. On the other hand, in both EarlyHyp and LateHyp clusters, women experienced a shorter time to occurrence of hypertension compared to men.

Table 2 presents the most frequent trajectories for each cluster. These trajectories were shared between men and women in the ELSA-Brasil cohort. Notably, within the FirstDM cluster, the most frequent trajectory for men was diabetes followed by hypertension (DM → HYP), while for women it was diabetes occurring in isolation. There was no significant difference between men and women concerning the percentage of the most frequent trajectories in each cluster. However, when considering the time to the occurrence, particularly within the FirstDM cluster, the DM→HYP trajectory showed a significantly shorter time to the onset of diabetes among women compared to men. In the UK Biobank cohort, most trajectories in each cluster were shared between men and women, except for two clusters. In the Healthy cluster, the second most frequent trajectory among women was standalone diabetes, while among men it was heart disease. Additionally, in the EarlyHyp cluster, the second most frequent trajectory for women was hypertension followed by diabetes (HYP→DM), whereas for men it was hypertension followed by heart disease (HYP→HD). There was no significant difference between men and women regarding the percentage of shared trajectories in each cluster, except for the Healthy cluster, where there were more women without diseases than men. Regarding the time of occurrence, specifically in the LateHyp and EarlyHyp clusters, women experienced hypertension in isolation earlier than men.

**Table 2:** Most frequent cardiometabolic continuum trajectories for each cluster according to sex and cohort

UKBiobank (Men, *N* = 8458)				UKBiobank (Women, *N* = 9242)			
	CMC Patterns				CMC Patterns		
Cluster	Pattern	Mean occurrence time	N (%)	Cluster	Pattern	Mean occurrence time	N (%)
EarlyHyp (*N* = 370)	HYP	0.23	343 (93)	EarlyHyp (*N* = 241)	HYP	0.19^+^	229 (95)
	HYP→*HD	0.19 →* 0.58	11 (3.0)		HYP→* DM	0.19 →* 0.69	5 (2.1)
FirstDM (*N* = 126)	DM → HYP	0.35 → 0.38	63 (50)	FirstDM (*N* = 101)	DM → HYP	0.36 → 0.39	49 (48.5)
	DM	0.34	48 (38)		DM	0.31	47 (46.5)
FirstHD (*N* = 199)	HD	0.32	93 (46.7)	FirstHD (*N* = 71)	HD	0.34	40 (56.3)
	HD→ HYP	0.38 → 0.41	89 (44.7)		HD → HYP	0.35 → 0.43	29 (40.8)
Healthy (*N* = 7313)	None Disease	—	7198 (98.4)	Healthy (*N* = 8490)	None Disease	—	8425 (99.2)^a^
	HD	0.7	39 (0.5)		DM	0.7	18 (0.2)
LateHyp (*N* = 450)	HYP	0.60	404 (89.8)	LateHyp (*N* = 339)	HYP	0.58^+^	318 (93.8)
	HYP→ HD	0.67→ 0.72	22 (4.8)		HYP→ HD	0.65→ 0.72	11 (3.2)
**ELSA-Brasil (Men**,***N* = 2870)**				**ELSA-Brasil (Women**,***N* = 4292)**			
	CMC Patterns				CMC Patterns		
Cluster	Pattern	Mean occurrence time	N (%)	Cluster	Pattern	Mean occurrence time	N (%)
EarlyHyp (*N* = 415)	HYP	0.23	354 (85)	EarlyHyp (*N* = 689)	HYP	0.23	601 (87)
	HYP→* DM	0.19 →* 0.74	31 (7.5)		HYP→* DM	0.20 →* 0.70	51 (7.4)
FirstDM (*N* = 81)	DM → HYP	0.41 → 0.43	42 (52)	FirstDM (*N* = 167)	DM	0.32	81 (48)
	DM	0.37	33 (41)		DM → HYP	0.35 → 0.48^+^	73 (43)
FirstHD (*N* = 88)	HD → HYP	0.28 → 0.32	42 (47.8)	FirstHD (*N* = 100)	HD→ HYP	0.30 → 0.42	55 (55)
	HD	0.24	32 (36.4)		HD	0.27	32 (32)
Healthy (*N* = 1981)	None Disease	—	1757 (88.7)	Healthy (*N* = 2837)	None Disease	—	2552 (89.9)
	HYP	0.88	102 (5.1)		HYP	0.88	151 (5.3)
LateHyp (*N* = 305)	HYP	0.61	271 (89)	LateHyp (*N* = 499)	HYP	0.62	452 (90)
	HYP→ DM	0.64→ 0.81	21 (7)		HYP→ DM	0.63→ 0.81	33 (6.6)

*→ Means ‘follow by’,^+^Difference between men and women regarding the pattern occurrence time, ^a^ Difference between men and women regarding the percentual. CMC: cardiometabolic continuum, HYP: hypertension, DM: diabetes mellitus, HD: heart diseases

Table 3 presents the performance measures for the multiclass random forest classifiers, adjusted by cohort and sex, considering the full set of predictors. The classifiers showed equivalent performances among sexes of the same cohort, with slightly better performance for the models fitted for women in the UK Biobank cohort (accuracy, precision, recall, and F1-score respectively equal to 0.64, 0.90, 0.63, and 0.73) and for men in the ELSA-Brasil cohort (mean accuracy, recall, and F1-score respectively equal to 0.57, 0.56, 0.51 and 0.52). In general, the models fitted to the UK Biobank data performed better.

**Table 3:** Multiclass random forest performance measures according to sex and cohort

	UKBiobank		ELSA - Brasil	
Performance Measures	Men	Women	Men	Women
Accuracy (mean, sd)	0.60 (0.01)	0.64 (0.02)	0.57 (0.05)	0.53 (0.04)
Precision (mean, sd)	0.80 (0.01)	0.90 (0.01)	0.56 (0.03)	0.54 (0.03)
Recall (mean, sd)	0.58 (0.02)	0.63 (0.05)	0.51 (0.06)	0.5 (0.03)
F1- score (mean, sd)	0.67 (0.02)	0.73 (0.03)	0.52 (0.03)	0.51 (0.03)

**Fig. 2:**
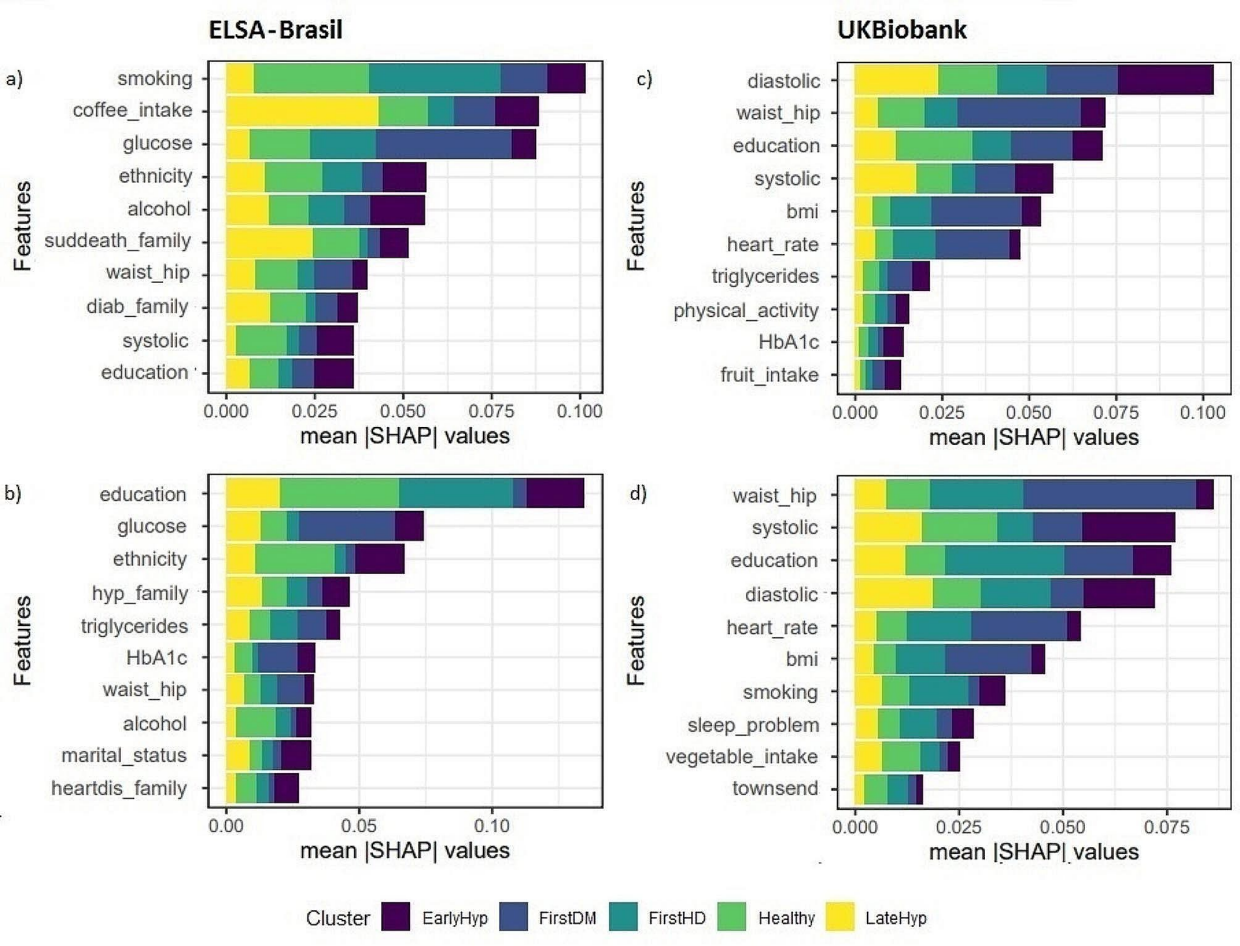
Average global variable’s importance for the first top 10 features using multiclass random forest by ELSA-Brasil (**a**) men and (**b**) women, and UKBiobank (**c**) men and (**d**) women. Suddeath_family: family history of sudden death, diab_family: family history of diabetes, hyp_family: family history of hypertension, heartdis_family: family history of heart disease, waist_hip: waist-hip ratio, townsend: UK Townsend deprivation index

**Fig. 3:**
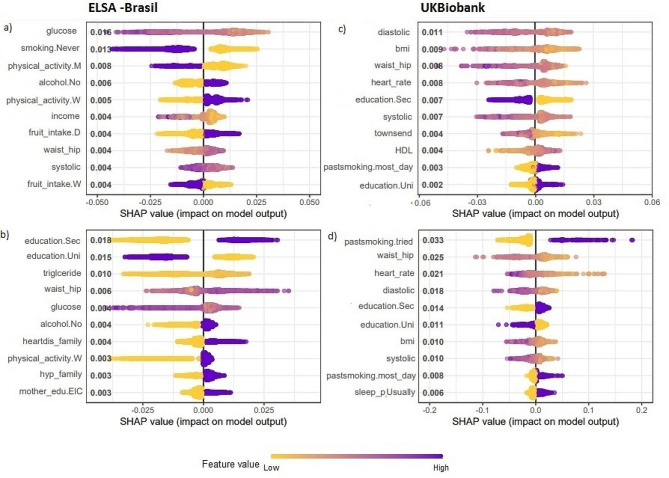
Local variable importance of the FirstHD cluster, based on the first top 10 features using multiclass random forest by ELSA-Brasil (**a**) men and (**b**) women, and UKBiobank (**c**) men and (**d**) women. Physical_activity.M: moderate physical activity, physical_activity.W: weak physical activity, fruit_intake.D: daily fruit intake, fruit_intake.W: weekly fruit intake, waist_hip: waist-hip ratio, education.Sec: secondary education, education.Uni: universit education, townsend: UK townsend deprivation index, HDL: HDL cholesterol level, hyp_family: family history of hypertension, heartdis_family: family history of heart disease, mother_edu.ElC: Complete elementary mother education, pastsmoking.most_day: past smoking most all days, sleep_p.Usually: usual sleep problem

To visualize the influence of the predictor variables on the classification of the different CMC patterns provided by the clusters, the SHAP values were used. Figure 2 shows the global importance ranking of the first 10 variables, according to the average of absolute SHAP values. For an in-depth analysis of the directionality of the most important variables, Fig. 3 and supplementary figures SFigs. 1, 2 and 3, and 4, in Appendix S1, depict the SHAP values of local importance (of each participant in the sample) for the 10 most important predictor variables within each cluster. In Fig. 3, the y-axis indicates the variable’s importance for classification, ranked from highest to lowest, while the x-axis shows the corresponding SHAP value. Each variable on the y-axis displays scattered points on the x-axis, representing individual observations of the participants. This means that for a given variable, the x-coordinate of a point indicates the SHAP value for the participant, that is the influence of the variable value on the participant’s classification within the cluster. Additionally, the colors of the points indicate the magnitude of the observed variable values, with yellow representing low values, and purple representing high values. This approach enables us to identify whether low values of the variable (yellow) negatively influence the classification probability (negative x-coordinate), leading to a decrease in the probability of classification within the cluster, or positively impact it (positive x-coordinate), resulting in an increase in the probability of classification within the cluster. Therefore, understanding both aspects of importance (global and local) is fundamental to truly understanding the effects of covariates on classification.

The most important cluster features differ based on the population and sex. The SHAP values for the UK Biobank showed that smoking and schooling were of great importance and had a clear directionality for both sexes. In ELSA-Brasil, education level and ethnicity had high importance and clear directionality for women, while for men these characteristics were smoking, alcohol consumption and coffee.

In the ELSA-Brasil cohort, education emerged as the variable of highest global importance for classifying women in all clusters, except for FirstDM, where glucose was the variable of highest global importance. University education decreased the probability of classifying women in the FirstHD and LateHyp clusters while increasing the probability of classification in the Healthy cluster. On the other hand, secondary education had the opposite influence. Self-reported white ethnicity positively influenced the classification of women in the Healthy cluster, but negatively influenced the classification in the EarlyHyp cluster. In contrast, non-white ethnicity had the opposite influence in these clusters. Diabetes in the family had a positive influence on the classification in the FistDM cluster for both sexes. For males, education did not hold the highest global importance in any cluster. Instead, smoking habits, alcohol consumption, and coffee consumption were among the variables with the greatest global importance. Never having smoked increased the probability of classification in the Healthy cluster but decreased in the FirstDM and FirstHD clusters. In the later cluster, moderate physical activity had a negative influence while low physical activity had a positive influence. Not drinking alcohol increased the likelihood of men’s classification in the EarlyHyp and FirstHD clusters, and drinking caffeinated coffee increased the likelihood of classification in the EarlyHyp, Healthy, and FirstDM clusters.

In the UK Biobank, the top 10 global importance variables showed more similarities between sexes than in the ELSA-Brasil cohort. Anthropometric measures, such as waist-hip, and BMI held higher importance for FirstDM classification, while blood pressure (systolic and diastolic), emerged as more influential variables for EarlyHyp and LateHyp classification, for both sexes. In the FirstHD cluster classification, women in the UK Biobank demonstrated similarities with the ELSA-Brasil, where education had the highest global importance. However, for the UK Biobank, even though continuous variables (anthropometric, blood pressure, and heart rate) had the greatest global influence, the directionality of their effects was not entirely clear. Regarding categorical variables, almost daily past smoking habits positively influenced the classification of both men and women in the FirstHD cluster. In contrast, university education had the opposite effects between the sexes, decreasing the probability of classification for women but increasing it for men. Secondary education positively influenced the FirstDM cluster but had a negative influence on the Healthy cluster for both sexes. For the EarlyHyp and LateHyp clusters, among males, never having smoked in the past and experiencing sleep problems rarely decreased the probability of classification, while secondary education increased it. Among women, the probability of classification was increased by almost daily past smoking and, in the EarlyHyp cluster, by university education and usual sleep problems.

## Discussion

The recent conceptualization of CMC calls for the investigation of the temporal relationships between CMDs and supports the need to consider the trajectory of diseases when defining prevention and intervention strategies that foster individualised care [1]. Traditional medical practices, which often focus on treating diseases in isolation, highlight the gaps and limitations faced by health systems, not including a generalist approach, and accessibility to the health system for patients who have been identified during the assessment as having complex care needs (clinically or in terms of social and socio-economic aspects) [14]. On the one hand, it is known that some risk factors have gender-dependent effects, as in the case of smoking, which doubles the risk of myocardial infarction in women compared to its impact in men. It is considered that CMD is not static; it changes with age, and its prevalence increases rapidly with age and gender. However, it is difficult to infer causality, so the list of causal risk factors is constantly changing. The main risk factors currently considered causal include total cholesterol, low-density lipoprotein (LDL) cholesterol and triglyceride levels; blood pressure; BMI; smoking; alcohol consumption; and type 2 diabetes. Some evidence of causality was also observed for lifestyle factors, such as diet quality, physical activity or stress [8–14]. Therefore, the formulation of effective health policies for CMDs should take into account sex, ethnicity, and population differences [8–10]. To the best of our knowledge, this is the first study to identify the temporal patterns of CMC trajectories and explore the sex differences across diverse populations in HICs and LMICs.

Lack of access to diverse cohorts and modeling complexity may explain the gap in the literature [19]. Incorporating the relationships between multiple CMDs in a single trajectory of occurrences is not an easy task. Addressing these challenges, recent studies have involved using multi-stage Markov models, which are limited to modeling the transitions along a trajectory in terms of changes between the healthy, first disease, and second disease states, without explicitly specifying which diseases occurred or integrating them into a trajectory [21, 28]. In this study, we present an intuitively simple and comprehensive machine learning approach that demonstrates the feasibility of modeling CMC trajectories by identifying temporal patterns and the factors that have the most influence on their occurrence. Notably, our approach extends beyond the specific context of CMC and has broad applicability in general scenarios involving the modeling of trajectories encompassing multiple diseases.

Our results showed five common patterns of CMC, identified for both sexes and cohorts through the following clusters: EarlyHyp, FirstDM, FirstHD, Healthy, and LateHyp. Despite these shared CMC patterns, important differences were found between the sexes, including variations in prevalence, time of disease occurrence, and importance of the predictors for classification, all of which were cohort-dependent. In the ELSA-Brasil cohort the highest proportion of women classified in the FirstDM cluster, as well as the highest isolated incidence of diabetes and hypertension among women, and heart disease among men, aligned with the Brazilian context described in health studies and surveys in recent years. The incidence rates of heart diseases including acute myocardial infarction, angina, and heart failure are higher among men, whereas the prevalence of hypertension and diabetes are higher among women, particularly those living in Brazilian capitals [29–31]. Our study adds an important result to this scenario: diabetes occurs as the first disease in the continuum more frequently among women. Furthermore, diabetes occurs faster among women who subsequently develop other diseases, particularly when hypertension becomes the next disease in the continuum. These findings emphasize the need for sex-specific interventions in the prevention and management of diabetes and cardiometabolic comorbidities, a requirement that has been highlighted in previous research [32–34]. Studies from different countries indicated that women with diabetes are currently less likely than men to receive treatment, and aimed at reducing the risk of cardiovascular disease. In Brazil, results from the Vigitel health survey between 2012 and 2019 indicated that the use of medications for diabetes control increased only among men [32–35].

However, it is worth noting that in most countries worldwide, the prevalence and incidence of diabetes and cardiometabolic diseases tend to be higher among men than among women, but it is important to acknowledge that these differences may vary depending on other factors, such as geographical region, ethnicity, age group, and socioeconomic variables [9, 33, 35–37]. Previous studies based on UK population data are consistent with this trend, and align with our findings [32, 36]. Overall, we observed that for the UK Biobank, the incidence of disease was higher among men than women, and this was reflected in the clusters since more men were classified in the clusters of participants with diseases and more women in the Healthy. Regarding the time of occurrence, a significant result emerged: women in the EarlyHyp and LateHyp clusters presented standalone hypertension occurring earlier than men. One plausible explanation could be that menopause in women is a period of expected transition from the observed epidemiological pattern of hypertension. While men have a higher prevalence until early midlife, women face the highest risk after menopause, and the onset of this period corresponds to the age range identified among women in the UK Biobank cohort [9, 36]. Thus, the lower time observed would favor the transition of the epidemiological pattern for hypertension among women.

According to the results for both cohorts, education plays an important role in the CMC pattern classification, except for men in the ELSA-Brasil cohort. Social vulnerability conditions, such as unemployment, low income, and low education, which in the Brazilian scenario are closely linked to ethnic and racial issues, have been previously identified as contributors to an increased risk of developing CMDs [8–10, 38]. In Brazil, this effect appears to be more pronounced among women than men [38]. Our results align with this direction, as high education levels increased the probability of classifying women from the ELSA-Brasil cohort into the Healthy cluster while decreasing the probability of classification into the LateHyp and FirstHD clusters. Additionally, white ethnicity self-reporting increased the probability of classifying women into the Healthy cluster, while decreasing the EarlyHyp cluster. Similarly, in the UK Biobank, the effect of education on CMC pattern classification was evident. Specifically, secondary education decreased the probability of classification in the Healthy cluster and increased the probability of classification in the FirstDM cluster for both sexes. It also positively influenced the classification probabilities for males in the EarlyHyp and LateHyp clusters. On the other hand, university education decreased the probability of classification in the FirstHD cluster for women while it increased for men, and had a positive influence on the classification of women in the EarlyHyp cluster. These last two results suggest that additional variables should be included to assess the influence of education on these two specific CMC patterns. Further exploration of pertinent factors could provide valuable insights into the complex relationship between education and CMC risk stratification for both cohorts.

Past smoking habits showed a notable influence on the classification of FirstHD patterns for both cohorts, except for women in ELSA-Brasil. Smoking has long been recognized as a significant risk factor for cardiovascular disease [39, 40]. Even after cessation, individuals who previously smoked continue to face an increased risk of developing cardiovascular diseases when compared with persons who never smoked [39, 40]. Consistent with the established literature, our results demonstrate that almost daily past smoking increased the probability of classification into the FirstHD cluster for both sexes in the UK Biobank. Additionally, among men in the ELSA-Brasil cohort, never having smoked negatively influenced the classification into the FirstHD, while positively influenced the classification into the Healthy cluster. In UK Biobank, smoking also influenced classification into the EarlyHyp and LateHyp clusters for both sexes with almost daily past smoking increasing the probability of classification for women and never having smoked decreasing this probability for men. Although the effects of smoking on blood pressure rise are not yet fully established, a possible relationship between prolonged blood pressure rise and masked hypertension in smokers has been reported [41].

The other predictors identified as being significantly relevant in predicting CMC patterns, such as sleep problems, alcohol and coffee consumption, and diabetes in the family, are the subject of investigation in studies on CMDs [42–47]. Sleep problems have been linked to blood pressure dysregulation and hypertension [42]. In line with these findings our results indicated that in the UK Biobank, women’s EarlyHyp classification was positively influenced by usual sleep problems and negatively influenced by sometimes experiencing sleep problems. For men, sleep problems never or rarely had a negative influence on both EarlyHyp and LateHyp clusters, and sometimes had a positive influence on the latter cluster. For ELSA-Brasil consistent with the literature, having diabetes in the family was one of the most positively influential variables affecting the classification into the FirstDM cluster for both sexes [43]. Drinking caffeinated coffee increased the likelihood of classification of men in the EarlyHyp, Healthy, and FirstDM clusters. The positive influence in both clusters with a healthy or diseased majority suggests an inconclusive effect in our study. Although studies have shown the potential benefit of moderate coffee consumption on CMDs prevention, other factors such as smoking could act as modifiers and must be taken into consideration [44, 45]. Furthermore, nonalcohol consumption increased the likelihood of men in the ELSA-Brasil cohort being classified into the EarlyHyp and FirstHD clusters. There is no established consensus in the literature about alcohol consumption. Although some studies have pointed out that the amount ingested needs to be evaluated, and moderate consumption may be beneficial, particularly in preventing CVDs, other studies suggest that alcohol consumption at any level increases the risk of hypertension and CVDs [46, 47].

Sex differences in the prevalence and onset time of CMC patterns, along with the set of most important individual characteristics, varied across the study populations, reflecting the large ethnic and socioeconomic diversity between Brazil and the UK, and confirming the need for sex and population-specific medical practices to address CMDs. Sex differences were more expressive in the ELSA-Brasil cohort than in the UK Biobank. This finding aligns with a 2019 review, where authors showed important differences between HICs and LMICs concerning CMDs and risk factors [12]. CVD deaths for example have decreased in recent decades in HICs, while increasing in LMICs, where a higher prevalence of these diseases is observed in younger populations. Moreover, although diabetes has increased worldwide, the rate of increase has occurred more rapidly in LMICs [12]. These differences may have a repercussion differentiated by sex, and be enhanced by multiple factors such as education, socioeconomic inequalities, as well as cultural and ethnic differences, making the trends observed between men and women more expressive in LMICs than HICs, as we observed in our study. A recent study indicates this direction, namely that CVD incidence and treatment are more expressive sex differences in LMICs than in HICs [13]. However, more studies are needed to understand these variations, validating the results in different populations and investigating the influence of other factors on CMC patterns, across the broad spectrum of CMDs.

The performance measures of the multiclass classifiers built for cluster prediction were influenced by the significant imbalance observed between the CMC patterns within both cohorts. Disparities in disease incidence, cluster imbalance, and the specific sets of predictors may have contributed to the observed variations in performance between the two cohorts [19, 48]. In addition, the lack of consistent directionality in the highest-ranked continuous variables possibly impacted the performance. These variables, in general, did not present clear directionality, with high values of the variables having both negative and positive influences on classification. A plausible explanation for this phenomenon could be attributed to a multidimensional and non-linear relationship between these predictors and the outcome identified by the ML classifiers, thus requiring a large number of observations to increase performance. This underscores the importance of using non-linear models to study CMC patterns, as they can better capture and interpret the complex relationship between variables.

Our study has some limitations. First, it is important to consider that the ELSA-Brasil cohort comprises public servants from educational and research institutions in Brazil, and thus may have relatively greater economic stability when compared to the Brazilian population. However, it is worth mentioning that the study was rigorously designed to capture the socioeconomic variability of the population enabling the detection of sex differences. Despite our results suggest possible CMC patterns in the populations of Brazil and the UK, further studies on this topic with population-based cohorts are therefore necessary. Another important factor that may have limited comparability was the fact that the set of predictors and diseases was not equal in the two cohorts. Some variables had different meanings, were either measured differently, or were not accessible in both cohorts. Moreover, the assessment times and follow-up were slightly different in the two cohorts, and although the age range was similar in both cohorts, the mean was different, indicating possible differences in age distribution. Finally, it should be mentioned that the importance of the variables found is limited to the model used (multiclass random forest classifier). The use of other classifiers can change the relevance of the variables. Furthermore, although SHAP is a valuable tool to study the influence and directionality of predictors, the causality relationship cannot be established. Thus, although it provides important insights about risk factors, it is not an appropriate technique for this purpose.

Despite these limitations, our study represents a pioneering effort to identify patterns of CMC trajectories, explore influential factors, and investigate differences between sexes and populations. In terms of prevention strategies, it is necessary to know not only which disease has the highest risk, but also which disease is most likely to be the first to occur to be able to define the first target of intervention. In this way, understanding CMC patterns can inform political decisions, favoring the allocation of resources and the development of more effective health programs. With the methodology we used for modeling CMC patterns, we show that it is possible to respond effectively to this problem. Furthermore, because the proposed modeling approach is simple, intuitive, and comprehensive, we demonstrate that it is possible to solve the modeling complexity faced by multi-occurrence disease trajectory studies. Thus, our study contributes valuable insights to the field of CMDs research and sets the stage for further exploration of tailored prevention and intervention strategies. By using a straightforward yet powerful methodology, we provide a foundation for resolving modeling complexities in future studies investigating multi-disease trajectories.

Our results revealed female disadvantage in terms of the time to onset CMDs. In the UK Biobank cohort, when hypertension is the first disease in the continuum and occurs in isolation, it occurs faster among women. In the ELSA-Brasil cohort, not only diabetes occurs more frequently as the first disease in the continuum among women, but it also occurs faster when followed by hypertension. Moreover, women had a higher incidence of hypertension and diabetes isolated, and a lower percentage of them were classified as healthy. Considering the inequitable access to suitable treatment and diagnosis for CMDs faced by women, our findings highlight the importance of sex-differentiated health policies in order to reduce existing inequalities, which are particularly more pronounced in Brazil.

### Electronic supplementary material

Below is the link to the electronic supplementary material.

Supplementary Material 1

## Data Availability

The datasets from ELSA-Brasil used and/or analysed during the current study are available from the corresponding author on reasonable request. The UK Biobank data used in this study can be accessed by applying through the UK Biobank Access Management System (www.ukbiobank.ac.uk/register-apply*).* This research was conducted using the UK Biobank re-source under Application 2964.
