# Supplementary material for: Sex and population differences in the cardiometabolic continuum: a machine learning study using the UK Biobank and ELSA-Brasil cohorts
Source: BMC Public Health. 2024 Aug 6;24:2131. doi: 10.1186/s12889-024-19395-9 (PMC11304673; doi:10.1186/s12889-024-19395-9)

Appendix S1

Contents

1. SChart 1: International Classification of Disease (ICD-10) codes for the UK Biobank conditions
2. SMethods 1: Average silhouette values according to the number of clusters
3. SMethods 2: Performance measures for the multiclass random forest classifier
4. STable 1: Sociodemographic characteristics for UKBiobank and ELSA-Brasil cohorts
5. STable 2: Incidence and occurrence time (in terms of follow-up fraction) of diseases, according to the sex and number of conditions developed during the follow-up.
6. STable 3: Incidence and occurrence time (in terms of follow up fraction) of diseases among the clusters.
7. SFig 1: Local variable importance of the FirstDM cluster.
8. SFig 2: Local variable importance of the EarlyHyp cluster
9. SFig 3: Local variable importance of the LateHyp cluster
10. SFig 4: Local variable importance of the Healthy cluster
11. SFig 5: Flow chart of study population (ELSA-Brasil)
12. SFig 6: Flow chart of UK Biobank population
13. SChart 1: International Classification of Disease (ICD-10) codes for the UK Biobank conditions

| **Disease** | **ICD - 10 Codes**  **Data field: f.41270** |
| --- | --- |
| Hypertension | I10, I11, I110,  I119, I12, I120,  I129, I13, I130, I131, I132, I139, I15, I150, I151,  I152, I158,  I159 |
| Diabetes | E10,E100, E101, E102, E103, E104, E105, E106, E107, E108, E109,   E11, E110, E111, E112, E113, E114, E115, E116, E117, E118,E119, E12, E120,E121, E122, E123, E124, E125, E126, E127, E128, E129,  E13, E130,E131, E132, E133, E134, E135, E136, E137, E138, E139,  E14, E140,E141, E142, E143, E144, E145, E146, E147, E148, E149 |
| Myocardial Infarction | I21, I210, I211, I212, I213, I214, I219, I222, I220, I221,I228, I229, I23, I230, I231, I232, I233, I234, I235, I236, I1238, I1241, I1252 |
| Angina | I20, I200, I201,  I208, I209, I24, I240, I248, I249 |
| Heart Failure | I50, I500, I501,I509 |
| Stroke | I60, I600, I601, I602, I603, I604, I605, I606, I607, I608, I609, I61, I610, I611, I612, I613, I614, I615, I616, I618, I619, I63, I630, I631, I632, I633, I634, I635, I636, I638, I639, I64 |

1. SMethods 1: Average silhouette values according to the number of clusters

|  | **Average silhouette values** | | | |
| --- | --- | --- | --- | --- |
|  | **UK Biobank** | | **ELSA -Brasil** | |
| Number of clusters | Men | Woman | Men | Woman |
| 2 | 0.87 | 0.93 | 0.74 | 0.73 |
| 3 | 0.86 | 0.93 | 0.69 | 0.76 |
| 4 | 0.89 | 0.93 | 0.73 | 0.73 |
| 5 | 0.91 | 0.95 | 0.75 | 0.78 |
| 6 | 0.89 | 0.94 | 0.72 | 0.76 |
| 7 | 0.90 | 0.94 | 0.75 | 0.75 |
| 8 | 0.91 | 0.95 | 0.74 | 0.75 |
| 9 | 0.89 | 0.94 | 0.72 | 0.76 |
| 10 | 0.91 | 0.94 | 0.75 | 0.74 |

1. SMethods 2: Performance measures for the multiclass random forest classifier

For model evaluation, we consider the performance measures usually considered for multiclass classifiers: Accuracy, precision, recall and F1-score. The metrics definition consider the following notation:

- y the set of true (sample, label) pairs
- $ŷ$ the set of predict (sample, label) pairs
- L the set of labels
- $y_{l}$ the subset of y with label l
- $\hat{y}_{l}$ the subset of $ŷ$ with label l
- $Ac\left( A,B \right)=\frac{|A\cap B|}{|A\cup B|}$, where A and B are sets, and | . | denotes the set cardinality
- $Pr\left( A,B \right)=\frac{|A\cap B|}{|B|}$
- $Re\left( A,B \right)=\frac{|A\cap B|}{|A|}$, when |A|=0 Re is denoted as 0, similarly for Pr and Ac.
- $F1\left( A,B \right)=2\frac{PrPr \left( A,B \right) * Re(A,B)}{PrPr \left( A,B \right) + Re(A,B)}$

The weighted metrics are defined as:

Accuracy: It is defined as the weigthed average of the proportion of correctly predicted labels concerning the total number of labels (predicted or observed) in each class.

$Accuracy=\frac{1}{\sum_{l \in L} \left| y_{l} \right|}\sum_{l \in L} \left| y_{l} \right| Ac(y_{l},\hat{y}_{l})$ (1)

Precision: The weigthed average of the precision (ability of the classifier not to label as positive a sample that is negative) in each class.

$Precision=\frac{1}{\sum_{l \in L} \left| y_{l} \right|}\sum_{l \in L} \left| y_{l} \right| Pr(y_{l},\hat{y}_{l})$ (2)

Recall: The weigthed average of the recall (ability of the classifier to find all the positive samples) in each class.

$Recall=\frac{1}{\sum_{l \in L} \left| y_{l} \right|}\sum_{l \in L} \left| y_{l} \right| Re(y_{l},\hat{y}_{l})$ (3)

F1-score: The weigthed average of the F1-score (harmonic mean of the precision and recall) in each class.

$F1\_score=\frac{1}{\sum_{l \in L} \left| y_{l} \right|}\sum_{l \in L} \left| y_{l} \right| F1(y_{l},\hat{y}_{l})$ (4)

1. STable 1: Sociodemographic characteristics for UKBiobank and ELSA-Brasil cohorts

| UKBiobank | | | | ELSA-Brasil | | | |
| --- | --- | --- | --- | --- | --- | --- | --- |
|  | Total  (N= 17700) | Men  (N=8458) | Woman (N=9242) |  | Total  (N= 7162) | Men (N=2870) | Woman (N=4292) |
| Age (years), mean (SD) | 55.4 (7.6) | 56.1 (7.7) | 54.8 (7.5) | Age (years), mean (SD) | 50.8 (7.4) | 50.4 (7.5) | 51 (7.3) |
| Education(n,%) |  | | | Education(n,%) |  |  |  |
| None | 1111 (6.3) | 544 (6.4) | 567 (6.1) | Elementary | 599 (8.4) | 351 (12.2) | 248 (5.8) |
| Secondary | 8227 (46.5) | 3776 (44.6) | 4451 (48.2) | Secondary | 2377 (33.2) | 930 (32.4) | 1447 (33.7) |
| University/ professional | 8362 (47.2) | 4138 (48.9) | 4224 (45.7) | University | 4186 (58.4) | 1589 (55.4) | 2597 (60.5) |
| Ethinicity (n,%) |  | | | Ethinicity (n,%) |  |  |  |
| White | 16126 (91.1) | 7778 (92) | 8348 (90.3) | White | 4053 (57.2) | 1609 (56.9) | 2444 (57.4) |
|  |  |  |  | Missing | 79 (1.1) | 43 (1.5) | 36 (0.8) |
| Townsendeprivation index, mean (SD) | -1.8 (2,8) | -1.9 (2.8) | -1.7 (2.9) | Income (per capita, US$) , mean (SD) | 1818.6 (1456.8) | 1717.2 (1399.9) | 1886.6 (1489.9) |
|  |  |  |  | Missing | 31 (0.4) | 11 (0.4) | 20 (0.5) |
| Live with others (Yes) (n,%) | 16697 (94.3) | 8370 (99) | 8327 (90.1) | Marital status, single (n,%) | 2449 (34.2) | 521 (18.2) | 1928 (44.9) |

1. STable 2: Incidence and occurrence time (in terms of follow-up fraction) of diseases, according to the sex and number of conditions developed during the follow-up.

| UKBiobank (N= 17700) | | | | | | | | | |
| --- | --- | --- | --- | --- | --- | --- | --- | --- | --- |
| Men (N=8458) | | | | | Woman (N=9242) | | | | |
|  | Number of conditions | | | |  | Number of conditions | | | |
|  | 1 (N=997) | | 2 or 3 (N=263) | |  | 1 (N=696) | | 2 or 3 (N=121) | |
| CMD | N, (%) | Time, mean (SD) | N, (%) | Time, mean (SD) | CMD | N, (%) | Time, mean (SD) | N, (%) | Time, mean (SD) |
| HYP | 764 (9) | 0.44 (0.22) | 254 (3)^a^ | 0.42 (0.23) | HYP | 564 (6.1)^b^ | 0.43 (0.23) | 119 (1.3)^a,b^ | 0.44 (0.23) |
| DM | 76 (0.9) | 0.46 (0.22) | 118 (1.4)^a^ | 0.45 (0.24) | DM | 65 (0.7) | 0.42 (0.23) | 68 (0.7)^b^ | 0.43 (0.22) |
| HD | 132 (1.6) | 0.43 (0.24) | 158 (1.9) | 0.46 (0.22) | HD | 52 (0.6)^b^ | 0.43 (0.23) | 54 (0.6)^b^ | 0.49 (0.23) |
| STK | 25 (0.3) | 0.48 (0.2) | 24 (0.3) | 0.55 (0.2) | STK | 15 (0.16) | 0.49 (0.21) | 8 (0.09) ^b^ | 0.43 (0.21) |
| ELSA - Brasil (N= 7162) | | | | | | | | | |
| Men (N=2870) | | | | | Woman (N=4292) | | | | |
|  | Number of conditions | | | |  | Number of conditions | | | |
|  | 1 (N=887) | | 2 or 3 (N=226) | |  | 1 (N=1427) | | 2 or 3 (N=313) | |
| CMD | N, (%) | Time, mean (SD) | N, (%) | Time, mean (SD) | CMD | N, (%) | Time, mean (SD) | N, (%) | Time, mean (SD) |
| HYP | 727  (25.3) | 0.47 (0.26) | 223 (7.8)^a^ | 0.42 (0.27)^*^ | HYP | 1204 (28)^b^ | 0.46 (0.26) | 306 (7.1)^a^ | 0.43 (0.28)^*^ |
| DM | 69 (2.4) | 0.61 (0.27) | 133 (4.6)^a^ | 0.63 (0.23) | DM | 144 (3.3)^b^ | 0.53 (0.28) | 199 (4.6)^a^ | 0.56 (0.26)^+^ |
| HD | 75  (2.6) | 0.54 (0.29) | 99 (3.5) | 0.49 (0.29) | HD | 62 (1.4)^b^ | 0.50 (0.28) | 129 (3.0)^a^ | 0.51 (0.27) |
| STK | 16 (0.6) | 0.65 (0.32) | 20 (0.5) | 0.60 (0.23) | STK | 17 (0.4) | 0.54 (0.28) | 18 (0.4) | 0.42 (0.30) |

^*^significant time difference between one disease and at least two disease subgroups, for the same sex. ^+^significant time difference between men and women considering the same number of conditions. ^a^significant incidence difference between one disease and at least two disease subgroups, for the same sex. ^b^significant incidence difference between men and women considering the same number of conditions. CMD: Cardiometabolic Diseases, HYP: Hypertension, DM: Diabetes Mellitus, HD: Heart Diseases, and STK: Stroke.

1. STable 3: Incidence and occurrence time (in terms of follow up fraction) of diseases among the clusters

| **ELSA-Brasil** (Men, N=2870) | | | | | | | | | | |
| --- | --- | --- | --- | --- | --- | --- | --- | --- | --- | --- |
|  | Clusters | | | | | | | | | |
|  | EarlyHyp  (N=415) | | FirstDM (N=81) | | FirstHD (N=88) | | Healthy (N=1981) | | LateHyp (N=305) | |
| CMD | N, (%) | Time, mean (SD) | N, (%) | Time, mean (SD) | N, (%) | Time, mean (SD) | N, (%) | Time, mean (SD) | N, (%) | Time, mean (SD) |
| HYP | 415 (100) | 0.23 (0.1) | 47 (58) | 0.43 (0.22) | 55 (62.5) | 0.32 (0.22) | 128 (6.5) | 0.88 (0.04) | 305 (100) | 0.62 (0.1) |
| DM | 35 (8.4) | 0.74 (0.14) | 81 (100) | 0.38 (0.16) | 11 (12.5) | 0.61 (0.24) | 54 (2.7) | 0.83 (0.1) | 21 (6.9) | 0.81 (0.09) |
| HD | 19 (4.6) | 0.75 (0.13) | 4 (4.9) | 0.74 (0.13) | 88 (100) | 0.24 (0.17) | 52 (2.6) | 0.77 (0.12) | 11 (3.6) | 0.77 (0.11) |
| STK | 11 (2.6) | 0.53 (0.25) | 2 (2.5) | 0.84 (0.18) | 3 (3.4) | 0.58 (0.36) | 18 (0.9) | 0.64 (0.31) | 2 (0.6) | 0.75 (0.16) |
| **ELSA-Brasil** (Woman, N= 4292) | | | | | | | | | | |
|  | Clusters | | | | | | | | | |
|  | EarlyHyp (N=689) | | FirstDM (N=167) | | FirstHD (N=100) | | Healthy (N=2837) | | LateHyp (N=499) | |
| CMD | N, (%) | Time, mean (SD) | N, (%) | Time, mean (SD) | N, (%) | Time, mean (SD) | N, (%) | Time, mean (SD) | N, (%) | Time, mean (SD) |
| HYP | 689 (100) | 0.22 (0.1) | 84 (50) | 0.45 (0.25) | 66 (66) | 0.42 (0.27) | 172 (6) | 0.88 (0.04) | 499 (100) | 0.63 (0.1) |
| DM | 54 (7.8) | 0.7 (0.17) | 167 (100) | 0.32 (0.15)* | 11 (11) | 0.62 (0.33) | 78 (2.7) | 0.82 (0.1) | 33 (6.6) | 0.81 (0.1) |
| HD | 28 (4) | 0.77 (0.12) | 11 (6) | 0.67 (0.23) | 100 (100) | 0.28 (0.14) | 41 (1.4)* | 0.77 (0.13) | 11 (2.2) | 0.78 (0.11) |
| STK | 9 (1.3) | 0.31 (0.25) | 2 (1) | 0.34 (0.47) | 2 (2) | 0.61 (0.1) | 19 (0.6) | 0.55 (0.29) | 3 (0.6) | 0.49 (0.35) |
| **UKBiobank** (Men, N=8458) | | | | | | | | | | |
|  | Clusters | | | | | | | | | |
|  | EarlyHyp (N=370) | | FirstDM (N=126) | | FirstHD (N=199) | | Healthy (N=7313) | | LateHyp (N=450) | |
| CMD | N, (%) | Time, mean (SD) | N, (%) | Time, mean (SD) | N, (%) | Time, mean (SD) | N, (%) | Time, mean (SD) | N, (%) | Time, mean (SD) |
| HYP | 370 (100) | 0.22 (0.12) | 77 (61.1) | 0.38 (0.2) | 99 (49.8) | 0.38 (0.2) | 22 (0.3) | 0.85 (0.05) | 450 (100) | 0.61 (0.11) |
| DM | 11 (2.9) | 0.62 (0.1) | 126 (100) | 0.34 (0.19) | 14 (7.0) | 0.57 (0.2) | 31 (0.4) | 0.7 (0.09) | 12 (2.7) | 0.72 (0.03) |
| HD | 13 (3.5) | 0.58 (0.14) | 13 (10.3) | 0.53 (0.15) | 199 (100) | 0.34 (0.18) | 43 (0.6) | 0.72 (0.09) | 22 (4.9) | 0.72 (0.04) |
| STK | 5 (1.3) | 0.26 (0.14) | 2 (1.6) | 0.45 (0.21) | 4 (2.0) | 0.62 (0.2) | 26 (0.3) | 0.49 (0.21) | 12 (2.7) | 0.64 (0.08) |
| **UKBiobank** (Woman, N=9242) | | | | | | | | | | |
|  | Clusters | | | | | | | | | |
|  | EarlyHyp (N=241) | | FirstDM (N=101) | | FirstHD (N=71) | | Healthy (N=8490) | | LateHyp (N=339) | |
| CMD | N, (%) | Time, mean (SD) | N, (%) | Time, mean (SD) | N, (%) | Time, mean (SD) | N, (%) | Time, mean (SD) | N, (%) | Time, mean (SD) |
| HYP | 241 (100) | 0.19 (0.11)* | 54 (53.4) | 0.4 (0.22) | 29 (40.8) | 0.43 (0.2) | 20 (0.2) | 0.83 (0.04) | 339 (100) | 0.59 (0.11)* |
| DM | 5 (2.1) | 0.69 (0.08) | 101 (100) | 0.34 (0.18) | 2 (2.8) | 0.49 (0.38) | 19 (0.2)* | 0.7 (0.07) | 6 (1.7) | 0.73 (0.05) |
| HD | 4 (1.6) | 0.63 (0.17) | 5 (4.9) | 0.52 (0.23) | 71 (100) | 0.34 (0.18) | 14 (0.2)* | 0.74 (0.06) | 12 (3.5) | 0.73 (0.04) |
| STK | 4 (1.6) | 0.36 (0.22) | 0 (0) | ____ | 0 (0) | ____ | 15 (0.2)* | 0.49 (0.21) | 4 (1.6) | 0.5 (0.21) |

^*^significant difference between men and women. CMD: Cardiometabolic Diseases, HYP: Hypertension, DM: Diabetes Mellitus, HD: Heart Diseases, and STK: Stroke.

1. SFig 1: Local variable importance of the FirstDM cluster, based on the first top 10 features using multiclass random forest by ELSA-Brasil a) men and b) women, and UKBiobank c) men and d) women.

SFig 1 shows that diabetes in the family increased the probability of classification in the First DM cluster for both sexes in the ELSA-Brazil cohort, and coffee consumption only for males. For the UK Biobank, secondary schooling increased the likelihood of classification in the FirstDM cluster for both sexes.


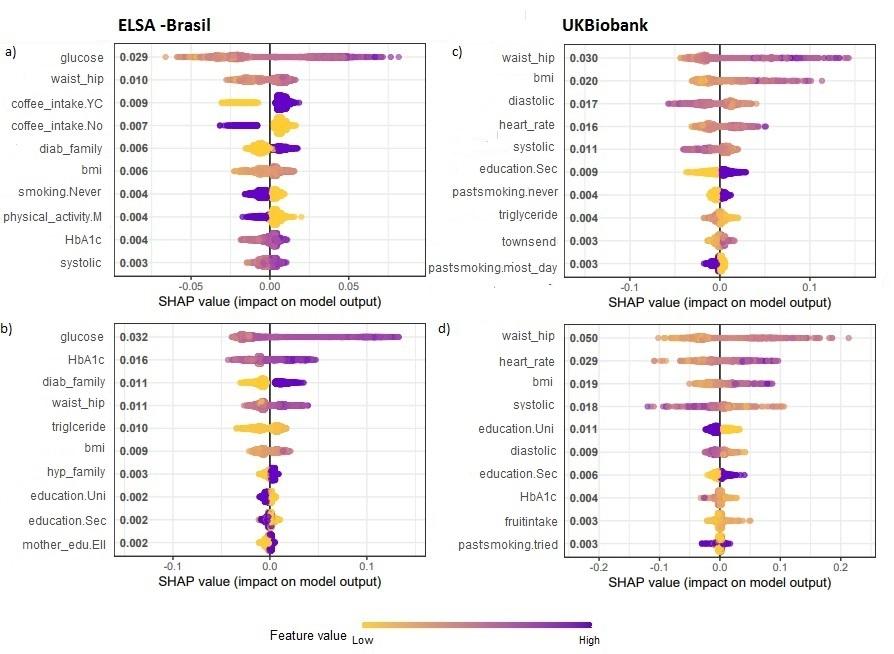


Physical_activity.M: moderate physical activity, coffee_intake.YC: caffeinated coffee intake, coffee_intake.No: No coffee intake, diab_family: family history of diabetes, HbA1c: glycated hemoglobin, waist_hip: waist-hip ratio, education.Sec: secondary education, education.Uni: university education, townsend: UK townsend deprivation index, pastsmoking.most_day: past smoking most all days, hyp_family: family history of hypertension, mother_edu.ElI: Incomplete elementary mother education.

1. SFig 2: Local variable importance of the EarlyHyp cluster, based on the first top 10 features using multiclass random forest by ELSA-Brasil a) men and b) women, and UKBiobank c) men and d) women.

Figure SFig 2 shows that for classification in the EarlyHyp cluster, differences between the sexes are observed for both cohorts. In the ELSA-Brazil cohort, moderate physical activity and coffee intake increased the likelihood of classifying men in the cluster, while elementary maternal education, non-white race/color and familial heart disease increased the likelihood of classifying women in the cluster. For the UK Biobank cohort, secondary education increased the probability of classification in the cluster for men, while women with university education, smoking almost every day and habitual sleep problems had an increased probability of classification.


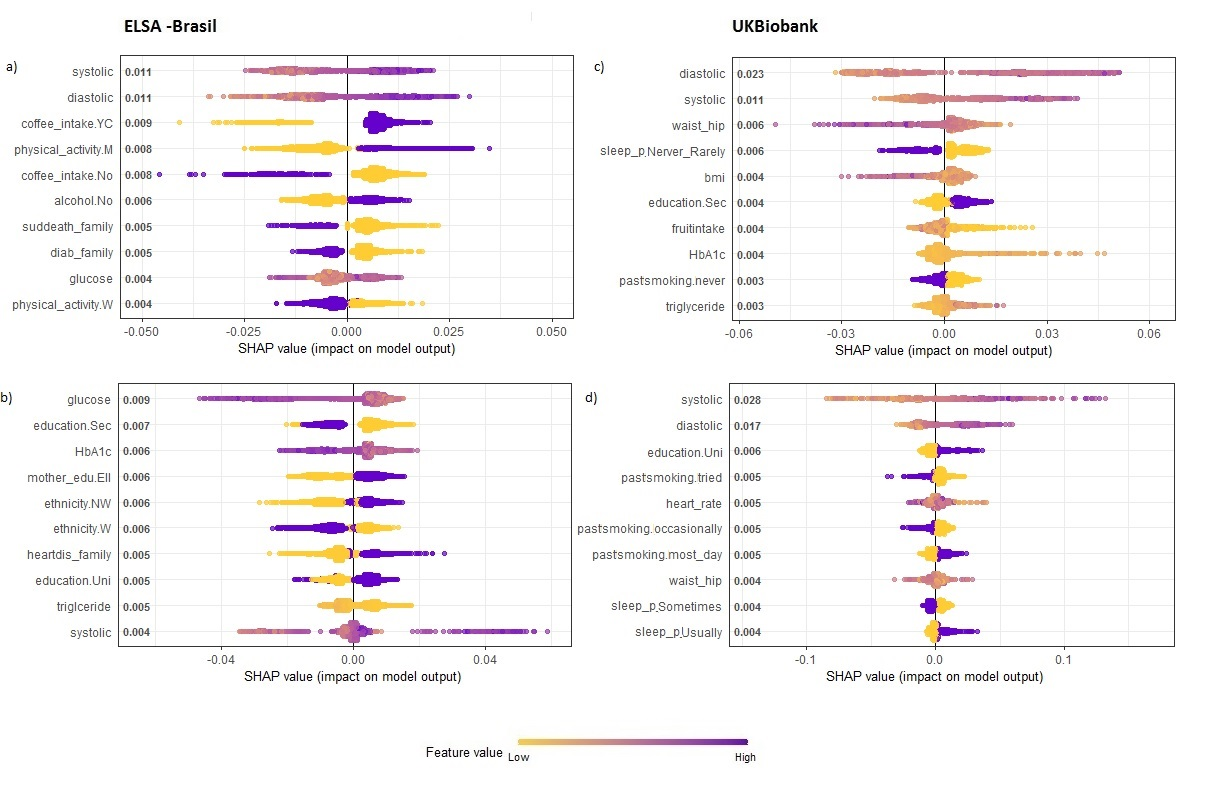


Physical_activity.M: moderate physical activity, physical_activity.W: weak physical activity coffee_intake.YC: caffeinated coffee intake, coffee_intake.No: No coffee intake, alcohol.No: No alcohol intake, diab_family: family history of diabetes, suddeath_family: family history of sudden death, heartdis_family: family history of heart disease, waist_hip: waist-hip ratio, sleep_p.Never_Rarely: never or rarely sleep problem, sleep_p.Usually: usual sleep problem, HbA1c: glycated hemoglobin, education.Sec: secondary education, education.Uni: university education, ethnicity.NW: non-white ethnicity, ethnicity.W: white ethnicity, pastsmoking.most_day: past smoking most all days, pastsmoking_tried: past smoking tried or twice, mother_edu.ElI: Incomplete elementary mother education.

1. SFig 3: Local variable importance of the LateHyp cluster, based on the first top 10 features using multiclass random forest by ELSA-Brasil a) men and b) women, and UKBiobank c) men and d) women.

SFig3 shows that for classification in the LateHyp cluster, there are differences between the sexes for both cohorts. In the ELSA-Brasil cohort, moderate physical activity and sudden death or diabetes in the family increased the probability of classifying men in the cluster, while elementary maternal education, secondary education and cancer in the family increased the probability of classifying women in the cluster. For the UK Biobank cohort, past habits of smoking almost every day increased the probability of classification in the cluster for both sexes, while secondary education increased the probability for men..


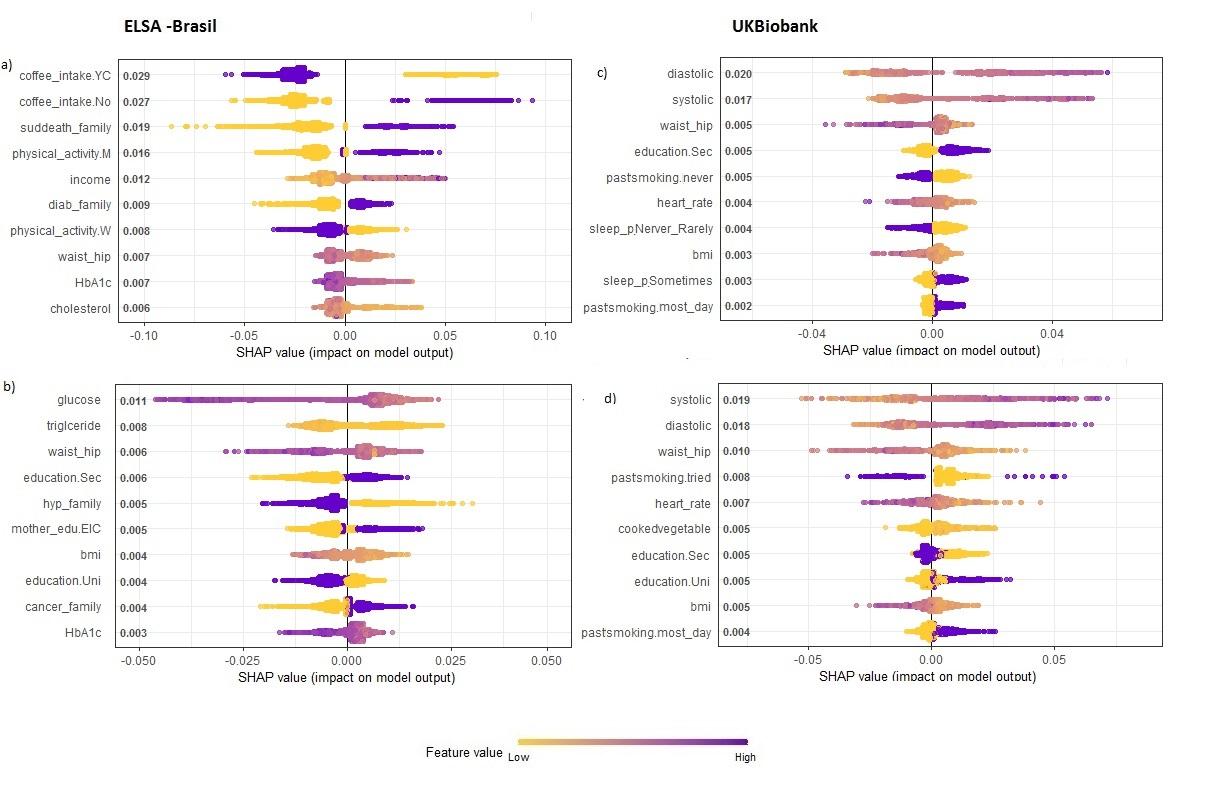


Physical_activity.M: moderate physical activity, physical_activity.W: weak physical activity coffee_intake.YC: caffeinated coffee intake, coffee_intake.No: No coffee intake, alcohol.No: No alcohol intake, diab_family: family history of diabetes, suddeath_family: family history of sudden death, hyp_family: family history of hypertension, cancer_family: family history of cancer, waist_hip: waist-hip ratio, sleep_p.Never_Rarely: never or rarely sleep problem, HbA1c: glycated hemoglobin, education.Sec: secondary education, education.Uni: university education, pastsmoking.most_day: past smoking most all days, pastsmoking_tried: past smoking tried or twice, mother_edu.ElC: Complete elementary mother education.

1. SFig 4: Local variable importance of the Healthy cluster, based on the first top 10 features using multiclass random forest by ELSA-Brasil a) men and b) women, and UKBiobank c) men and d) women.

Figure SFig 4 shows that for classification in the Healthy cluster, differences are observed between the sexes for both cohorts. In the ELSA-Brazil cohort, coffee intake and never having smoked increased the probability of classification for men in the cluster, while university education and white race increased the probability of classification for women in the cluster. For the UK Biobank cohort, university education and not having a habitual sleep problem increased the likelihood of classification in the cluster for men and, for women, the likelihood of classification was decreased by having ever tried smoking in the past.


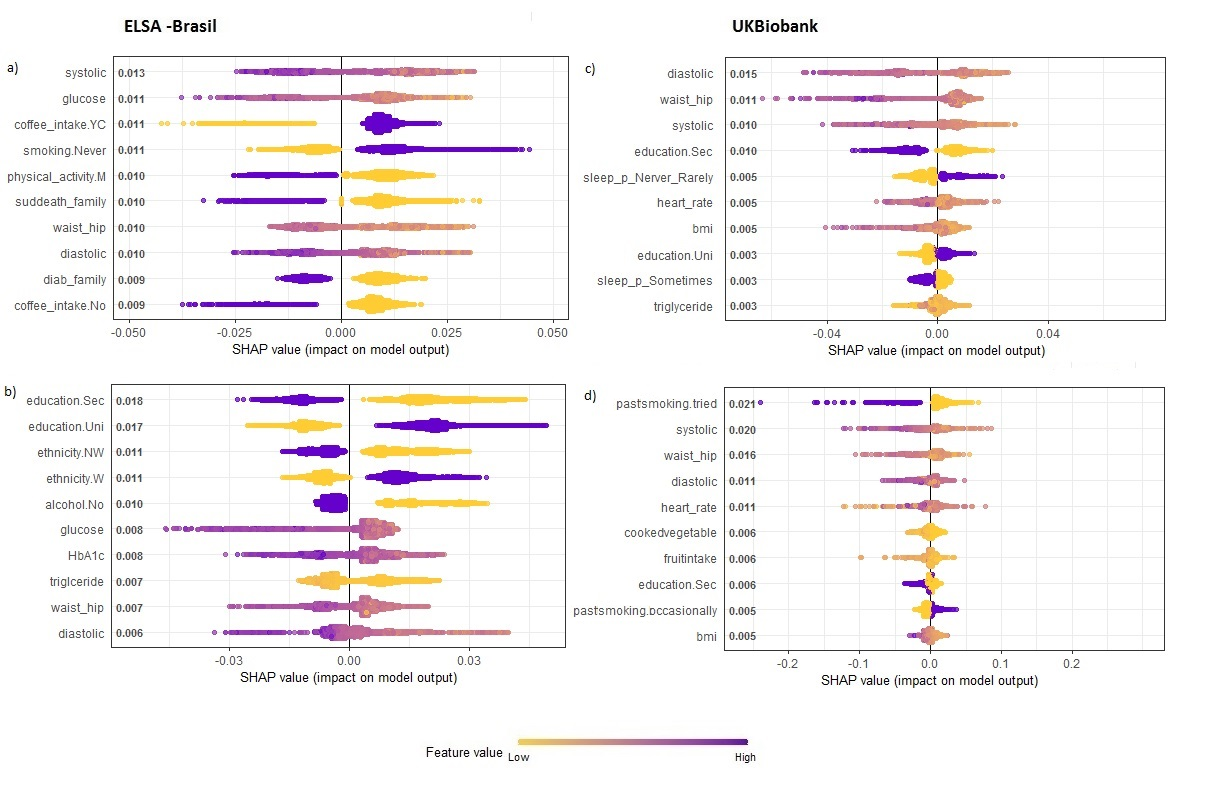


Physical_activity.M: moderate physical activity, coffee_intake.YC: caffeinated coffee intake, coffee_intake.No: No coffee intake, alcohol.No: No alcohol intake, diab_family: family history of diabetes, suddeath_family: family history of sudden death, waist_hip: waist-hip ratio, sleep_p.Never_Rarely: never or rarely sleep problem, HbA1c: glycated hemoglobin, ethnicity.NW: non-white ethnicity, ethnicity.W: white ethnicity, education.Sec: secondary education, education.Uni: university education, pastsmoking_tried: past smoking tried or twice.

11 - SFig 5: Flow chart of study population (ELSA-Brasil)


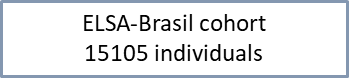


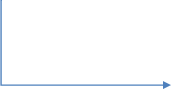

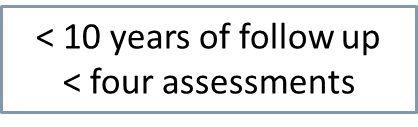

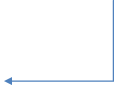


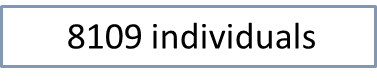


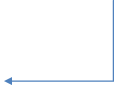

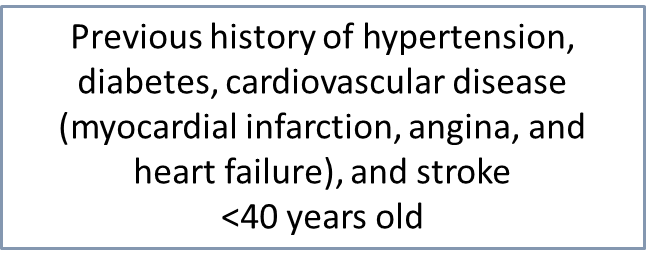

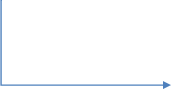


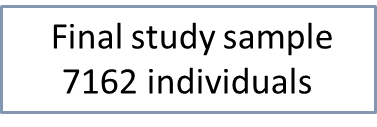


12 - SFig 6: Flow chart of UK Biobank population


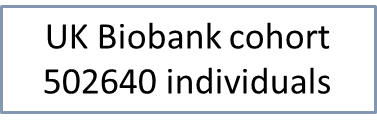


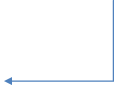

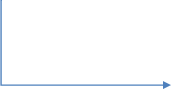

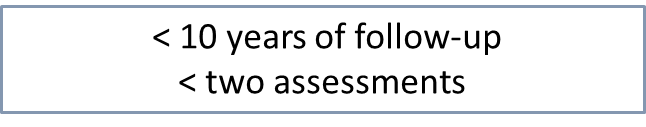


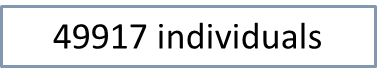


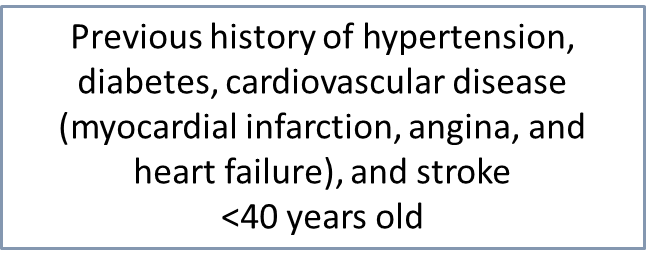

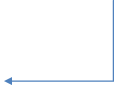

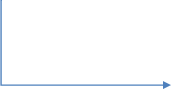


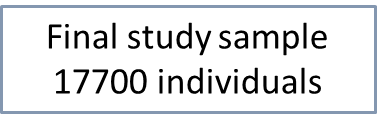

Supplement: Supplementary file 1 — Supplementary Material 1 [file 12889_2024_19395_MOESM1_ESM.docx]
